# Prevalence and diversity of Acanthocephala in stream-dwelling amphipods (*Gammarus fossarum*) around an urban area in the eastern Alpine foothills

**DOI:** 10.1017/S0031182025100449

**Published:** 2025-06

**Authors:** Fabian Gallhammer, Jacqueline Grimm, Susanne Reier, Kristina M. Sefc

**Affiliations:** 1 Institute of Biology, University of Uni Graz, Graz, Austria; 2 First Zoological Department, Natural History Museum Vienna, Vienna, Austria

**Keywords:** Acanthocephala, Amphipoda, aquatic parasites, barcoding, bioindicator, COI sequences, environmental impact, Oxford nanopore sequencing, prevalence, stream habitat

## Abstract

Population dynamics of aquatic parasites respond to factors like host availability, habitat age and quality. Amphipods are intermediate hosts for Acanthocephala, a widespread group of parasitic worms. Acanthocephalan infections of amphipods can easily be detected, and the widespread occurrence of amphipods makes their infection status an attractive potential proxy for the ecological status of their aquatic environment, including stressors introduced by urbanization. This study investigated the prevalence and the species-level and genetic diversity of Acanthocephala in the stream amphipod *Gammarus fossarum*. The study streams cross forested, agricultural and urban landscapes in the eastern foothills of the European Alps. Parasite prevalence ranged from 0% to 8.8% and increased towards downstream reaches independent of surrounding land use. Oxford Nanopore Technology was used to sequence the mitochondrial cytochrome oxidase I barcoding locus to identify parasite species and assess their genetic diversity. The majority of the parasites were *Pomphorhynchus tereticollis*, which use fish as definitive hosts. Despite their relative abundance in the studied streams, their genetic diversity was low and the most common haplotype was found at all sampling sites, which might indicate population expansion. Amphipods also hosted *P. laevis* and *Polymorphus* sp. type 1, the first evidence of this cryptic species within *Polymorphus* cf. *minutus* in Austria. Genetic diversity was high in *Polymorphus* sp. type 1, possibly reflecting a large effective population size due to gene flow maintained by the avian final hosts. The low and downstream-biased prevalence suggests that definitive hosts may be a limiting factor for Acanthocephala populations in small streams.

## Graphical abstract

**Figure:**
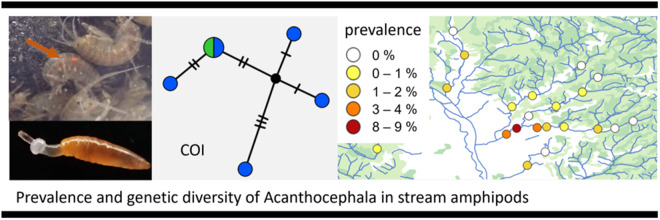

## Introduction

Population dynamics of parasite species are influenced by multiple internal and external conditions and population sizes of a given parasite species can therefore vary considerably in space and time. One obvious potential driver of parasite population dynamics is the availability and abundance of suitable hosts (Arneberg et al., 1998; Kmentová et al., 2016; Song and Proctor, 2020). Habitat age and stability, or time since colonization, are further potential drivers (Song and Proctor, 2020). In addition to natural influences, parasites also respond to anthropogenic stressors, which has led to investigations regarding the usefulness of parasite abundance as bioindicator of various types of pollution (Poulin, 1992; Lafferty, 1997; Blanar et al., 2009; Vidal-Martinez et al., 2010; Sures et al., 2017; Marcogliese, 2023). The effects of pollution on parasite abundance can be positive, e.g. when environmental stress increases host susceptibility, or negative, when environmental stressors weaken the parasites themselves (Blanar et al., 2009; Marcogliese, 2023). In a recent study, the prevalence of acanthocephalan parasites in their intermediate amphipod hosts was positively correlated with local pollution levels along an anthropogenically impacted river in southern France (Fanton et al., 2022). Conversely, the abundance of acanthocephalans in the definitive fish host was negatively correlated with pollution levels in a southern Brazilian river (Lacerda et al., 2018).

Acanthocephalans are endoparasitic worms with complex life cycles involving at least one arthropod intermediate and one vertebrate definitive host (Kennedy, 2006). Intermediate hosts of Acanthocephala in European freshwater systems are amphipods and aquatic isopods, while definitive hosts differ between acanthocephalan genera. Species of *Pomphorhynchus* and *Echinorhynchus*, for example, complete their life cycle in fish, whereas birds serve as final hosts for *Polymorphus* spp. The mature worms reproduce in the digestive tract of their vertebrate hosts and their eggs are shed into the water with the feces. The intermediate hosts are then infected by ingesting the parasite eggs. The parasite larvae hatch in the intestines and develop in the body cavities of the intermediate hosts until they reach the infective stage for the definitive host (Sures and Schmidt-Rhaesa, 2014). Several acanthocephalan species are known to manipulate the behaviour of their intermediate hosts to enhance trophic transmission to the definitive host (Bakker et al., 2017).

Common acanthocephalan species found in Central Europe are *Polymorphus minutus, Pomphorhynchus laevis, Pomphorhynchus tereticollis* and *Pomphorhynchus bosniacus* (Ladewig et al., 2006; Westram et al., 2011; Bauer and Rigaud, 2015; Galipaud et al., 2019; Reier et al., 2019, 2020; Jirsa et al., 2021; Fanton et al., 2022). Genetic data revealed considerable intraspecific genetic diversity and, in some of these species, also geographic structure across Europe (Smrzlić et al., 2015, 2024; Perrot-Minnot et al., 2018; Zittel et al., 2018; Reier et al., 2019, 2020, Jirsa et al., 2021). *Polymorphus minutus* has been shown to encompass three morphologically cryptic but genetically divergent species, separated by 3.5–11% distance in cytochrome oxidase I (COI) sequences, each of which was associated with a different amphipod host species (Zittel et al., 2018). The cryptic species are widely distributed in Germany and France (Zittel et al., 2018), and one of them (*Polymorphus* sp. type 3) has meanwhile also been recorded further east, in Austria (Jirsa et al., 2021). Somewhat lower levels of genetic divergence were detected in *P. laevis* (mean 2%, max. 3.5%, COI) and *P. tereticollis* (mean 1.8%, max. 3.8%, COI), with *P. tereticollis* showing a weak phylogeographic structure comprising four haplogroups across Europe (Reier et al., 2019). *P. laevis* contains two clearly distinct haplogroups (Eastern and Western European), of which only the Eastern European haplogroup was detected in Austria (Reier et al., 2019). The extent of intraspecific genetic diversity and population and phylogeographic structure present in Acanthocephala is expected to reflect host specificity, mobility of the host and demographic history of the parasite populations (Song et al., 2014; García-Varela et al., 2023; Sromek et al., 2023), but population level data on genetic diversity in Acanthocephala are still rather scarce.

The potential ecological consequences of Acanthocephala infections of amphipods are diverse. Amphipod population and invasion dynamics can be altered by parasite-induced effects on predation risk, overall mortality, competitive ability, sensitivity to pollutants and other environmental stressors (Giari et al., 2020; Kochmann et al., 2023). On an even broader scale, parasite-induced modifications of how amphipods interact with their biotic and abiotic environments can have implications for entire aquatic ecosystems (Giari et al., 2020), for instance due to the reduced shredding and increased bioturbation activity of infected amphipods (Labaude et al., 2017; Williams et al., 2019).

**Figure 1.**
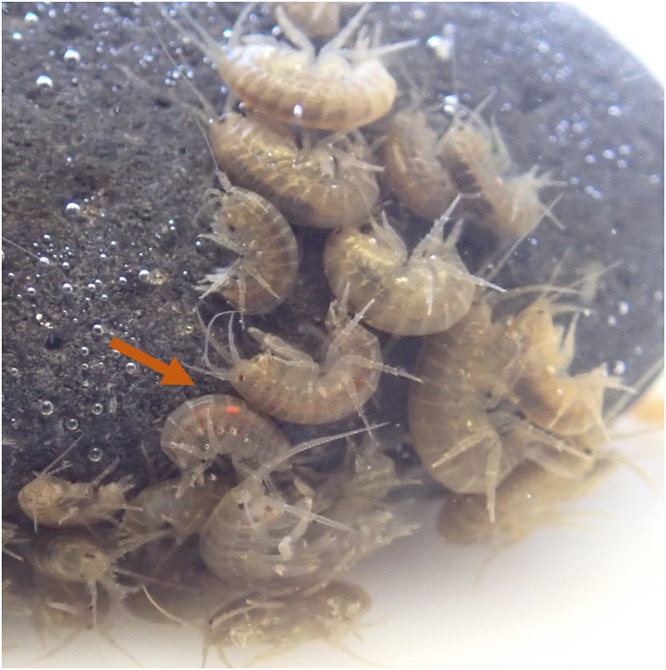
A group of *Gammarus fossarum*, one of which is visibly infected with an orange-coloured acanthocephalan (arrow). Photo: K. M. Sefc.

The abundance and widespread occurrence of amphipods in streams and rivers also makes their infection status an attractive potential proxy for the ecological status of their aquatic environment, especially considering that Acanthocephala larvae can be detected with the naked eye through the amphipod cuticle (Figure 1). However, while the idea to use acanthocephalans in environmental monitoring has been brought up repeatedly (Lafferty, 1997; Fanton et al., 2022; Perrot-Minnot et al., 2023; Sures et al., 2023), the relationships between environmental stressors and Acanthocephala abundance are not investigated exhaustively (Blanar et al., 2009; Lacerda et al., 2018; Fanton et al., 2022). Clearly, the prevalence of acanthocephalan parasites in European streams and rivers varies depending on study site, parasite and host species (Ladewig et al., 2006; Westram et al., 2011; Bauer and Rigaud, 2015; Galipaud et al., 2019; Fanton et al., 2022), and more data are needed to evaluate the potential factors at various geographic scales. In the present study, we focus on several small streams that originate in the hills around the city of Graz (Styria, Austria) and cross forested, agricultural and urban landscapes before flowing into the River Mur. During previous amphipod collections, we had already noticed acanthocephalan infections of amphipods in this region, particularly at sites in the more developed, urban area. Urban stream sections are typically surrounded by artificial, impervious surfaces; are hydromorphologically impacted by stream bed modifications; and are exposed to elevated pollution due to, e.g. road run-off (Allan, 2004). Relationships between fish parasite abundances and degrees of urbanization have been demonstrated in both freshwater and marine systems (Hernandez et al., 2007; Taglioretti et al., 2018; Shah Esmaeili et al., 2021). To examine whether spatial variation in acanthocephalan prevalence was associated with the urban environment, prevalence estimates in urban and residential stream sections were compared with stream sections traversing the surrounding rural landscape, which is characterized by agricultural land and forests.

Furthermore, we assessed species diversity and intraspecific genetic diversity of Acanthocephala in the study region. To this aim, the acanthocephalan larvae were extracted from their amphipod hosts and identified to species level using DNA barcoding (Hebert et al., 2003). We then used the DNA sequence data to estimate genetic diversity in the parasite populations, and joined the new sequences with published datasets to relate the local diversity in our study region to the phylogeographic patterns across the distribution ranges.

## Material and methods

### Parasite prevalence

**Figure 2.**
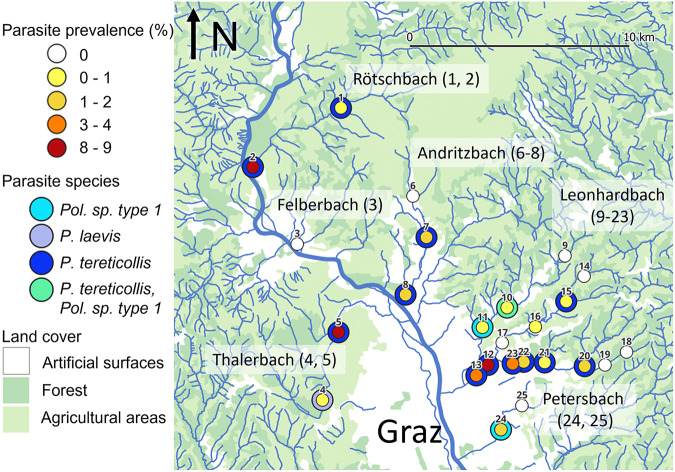
Acanthocephala prevalence and species at the study sites. Study sites are numbered from 1 to 25 as in Table 1. The colour of the inner circle represents the prevalence category, and the colour of the outer ring represents the parasite species found at the site (if any). The River Mur is drawn in a thick line, and the six tributary stream systems are named according to the stream name at the confluence with the Mur, with the corresponding site numbers in parentheses. The map was created in QGIS 3.42.2, using the European Union’s Copernicus Land Monitoring Service information (https://doi.org/10.2909/71c95a07-e296-44fc-b22b-415f42acfdf0) and GIS stream data provided by the Umweltbundesamt GmbH (https://docs.umweltbundesamt.at/s/8rYyRjFDse8Za4D).

*Gammarus fossarum* were collected from 25 sampling sites in small streams in and around the city of Graz, Austria, located in the eastern foothills of the European Alps (Figure 2, Tables 1 and S1). The sampling sites comprised five stream drainage systems which discharge into the river Mur (Figure 2, Table 1). Land use around the sampling sites varied and included built-up areas in the city, as well as agricultural and forested areas in the periphery. Stream widths at the sampling sites ranged from 0.5 to 3 m and water depths ranged from 10 to 30 cm. To assess temporal variation in parasite prevalence, one site (#12) was sampled once a month from April to August 2023, and once more in October 2023 (Table S2). The other sites were sampled between mid-April and early July 2023. Amphipods were collected by disturbing the stream bed and detritus patches, upon which the animals were flushed into hand nets by the water current. Sampling effort was not standardized across sites, but we aimed at sample sizes of 100 or more amphipods to allow the estimation of low infection prevalence. All amphipods collected in the net were taken to the laboratory to estimate parasite prevalence, and zoobenthos other than amphipods were returned to the stream. Temperature, conductivity and oxygen concentration and saturation were measured at sampling sites using a handheld multiparameter probe (pHenomenal MU 6100H by VWR).

**Table 1.** Sampling sites and acanthocephalan parasitism

Site	Stream system (stream)	Land use	Cond. (μS cm^−1^)	Sample size	No. inf.	Prevalence (%)	Parasite species	No. *P*_(seq)_	*G. fossarum* MOTUs
1	Rötschbach	agr/for	417	437	2	0.05	P. t.	2	SEE3, SEE5, (SEE1)
2	Rötschbach	agr/for	405	177	15	8.47	P. t.	10	SEE5
3	Felberbach	urb/res	558	796	0	0.00	NA	NA	(SEE5)
4	Thalerbach (Katzelbach)	agr/for	469	434	1	0.23	P. l.	1	SEE5
5	Thalerbach	agr/for	516	658	58	8.81	P. t.	39	SEE5
6	Andritzbach	urb/res	468	338	0	0.00	NA	NA	(CEE1, SEE3)
7	Andritzbach	urb/res	437	518	8	1.54	P. t.	8	SEE3
8	Andritzbach	urb/res	437	526	8	1.52	P. t.	8	SEE3, (SEE1)
9	Leonhardbach (Föllingerbach)	agr/for	508	870	0	0.00	NA	NA	(SEE2)
10	Leonhardbach (Mariatrosterbach)	urb/res	512	306	3	0.98	PspT1, P. t.	3	SEE2
11	Leonhardbach (Mariatrosterbach)	urb/res	504	403	4	0.99	PspT1	3	SEE2
12	Leonhardbach	urb/res	447	612	49	8.01	P. t.	28	SEE2
13	Leonhardbach	urb/res	494	365	12	3.29	P. t.	12	SEE2
14	Leonhardbach (Stiftingbach)	agr/for	337	745	0	0.00	NA		(SEE2)
15	Leonhardbach (Stiftingbach)	agr/for	429	640	5	0.78	P. t.	5	SEE2
16	Leonhardbach (Stiftingbach)	urb/res	398	273	1	0.37	P. t.	1	SEE2
17	Leonhardbach (Stiftingbach)	urb/res	418	395	0	0.00	NA	NA	(SEE2)
18	Leonhardbach (Ragnitzbach)	agr/for	130.6	92	0	0.00	NA	NA	(SEE2)
19	Leonhardbach (Ragnitzbach)	agr/for	323	208	0	0.00	NA	NA	(SEE2)
20	Leonhardbach (Ragnitzbach)	urb/res	342	413	5	1.21	P. t.	3	SEE2
21	Leonhardbach (Ragnitzbach)	urb/res	416	422	2	0.47	P. t.	2	SEE2
22	Leonhardbach (Ragnitzbach)	urb/res	469	253	5	1.98	P. t.	5	SEE2
23	Leonhardbach (Ragnitzbach)	urb/res	474	385	12	3.12	P. t.	5	SEE2
24	Petersbach	urb/res	519	159	2	1.26	PspT1	1	SEE2
25	Petersbach	urb/res	545	400	0	0.00	NA	NA	(SEE2)

Site numbering and stream systems correspond to Figure 2; stream names that differ from the name of the stream system are given in parentheses. ‘Sample size’ refers to the number of examined amphipods, and ‘No. inf.’ is the number of infected amphipods. Land use is scored as agricultural and forest (‘agr/for’) and urban and residential (‘urb/res’). Abbreviations for parasite species are as follows: P. t., *Pomphorhynchus tereticollis*; P. l., *Pomphorhynchus laevis*; PspT1, *Polymorphus* sp. type 1 (Zittel et al., 2018). ‘No. P(seq)’ indicates the number of Acanthocephala individuals for which COI barcode sequences were obtained. Conductivity (cond.) was measured at the time of amphipod sampling. *G. fossarum* MOTUs identified at the study sites are indicated by clade (SEE or CEE, sensu Wattier et al., 2020) and an internal lab number; MOTUs that were not infected by Acanthocephala are written in parentheses. Further information on sampling sites is given in Table S1.

*Gammarus fossarum* was the only amphipod species present at the study sites. Sample sizes ranged from 92 to 870 amphipods per site (mean = 432.8, median = 403; Table 1). In the laboratory, visibly infected amphipods (based on orange acanthella or cystacanth stages that could be detected through the host cuticle) were separated from non-infected amphipods and euthanized in carbonated water. Infected amphipods were counted and individually weighed on a laboratory scale. Then, the acanthocephalan larvae were extracted from their hosts and preserved in 99% ethanol for DNA barcoding. With few exceptions, each host contained a single parasite larva (at the high-prevalence site #5, 2 host individuals each contained 4 larvae). The host tissues were stored separately.

The uninfected amphipods were also euthanized in carbonated water. Three of the smallest and three of the largest individuals were weighed individually to estimate the weight range, and the remaining individuals were weighed in bulk. The mean weight of uninfected amphipods was determined based on the sum of the individual and bulk weights. The number of uninfected amphipods was determined by automatic counting. To do this, the amphipods were spread out on a semi-translucent surface placed on a visible light transilluminator, making sure that individuals did not touch each other, and photographed. The ‘Analyze Particles’ function of ImageJ was used to count the amphipods on the photos.

Acanthocephala prevalence was calculated as the proportion of infected amphipods in the sample collected at each site. To examine potential drivers of spatial variation in Acanthocephala prevalence, we tested for correlations with land cover, conductivity and distance from source. Land cover in a 100 m radius around each site was determined based on the CORINE land cover 2018 dataset (using the European Union’s Copernicus Land Monitoring Service information, https://doi.org/10.2909/71c95a07-e296-44fc-b22b-415f42acfdf0) and classified as urban and residential areas (CORINE class ‘artificial surfaces’) or natural surface (combining the CORINE classes ‘agricultural areas’ and ‘forest and seminatural areas,’ which alternate at small spatial scales in the study area). Conductivity measures the presence of ions (e.g. chloride, sulphite and nitrate) in the water and is related to water quality (Harwell et al., 2008; Thompson et al., 2012; Skarbøvik and Roseth, 2015) and the ecological status of water bodies (Potapova and Charles, 2003; Zampella et al., 2006). Land cover and conductivity represented variation in environmental conditions, which might be associated with variation in environmental quality. Distance from source reflects the locations of the sampling sites in upper, middle and lower parts of the streams and might be connected to parasite prevalence in amphipods, if Acanthocephala eggs shed by the definite hosts accumulated downstream. Distances between sampling sites and the source of the respective streams along the stream course were calculated using the GIS stream layer provided by the Umweltbundesamt GmbH (https://docs.umweltbundesamt.at/s/8rYyRjFDse8Za4D). We designated the farthest upstream spring outlet as source point for this calculation. We fitted a general linear model (GLM) with a negative binomial error distribution (R package MASS, function glm.nb; Venables and Ripley, 2022), including the number of infected amphipods as response variable. To account for differences in the total number of amphipods that were collected per site, sample size was included as offset variable. Model predictors were surrounding land cover (nominal variable with 2 levels: ‘artificial surface’, i.e. urban and residential areas; and ‘natural surface’, i.e. agricultural, forest and seminatural areas), conductivity (metric variable, in μS cm^−1^) and distance from source (metric variable, in kilometres). An alternative model (overdispersed binomial logit model; R package dispmod, function glm.binomial.disp; Scrucca, 2018) yielded qualitatively identical results. Collinearity among predictors was measured by the variance inflation factor (VIF) calculated in the R package ‘car’ (Fox and Weisberg, 2019). VIFs of 1 indicate no collinearity, while VIFs >5 indicate high collinearity between predictors and coefficients cannot be estimated reliably. For site #12 (which had been sampled repeatedly), we summed the numbers of infected and uninfected amphipods collected in April, May and June, and took the mean of the conductivity and oxygen saturation values measured at these time points. Omitting site #12, covariation between parasite prevalence and sampling time was tested in a separate GLM, with ‘day’ (starting with 0 for the first day of sampling) as predictor.

### DNA barcoding and genetic diversity

DNA was extracted from whole Acanthocephala larvae using a standard Chelex protocol (Richlen and Barber, 2005). The DNA barcode region of the COI gene was amplified using primers LCO1490-JJ2 (5ʹ-CHACWAAYCAYAARGAYATYGG) and HCO2198-JJ2 (ANACTTCNGGRTGNCCAAARAATCA) (Astrin et al., 2016). The polymerase chain reactions (PCRs) contained 0.35 µL of a 2.5 mM dNTP mix, 0.5 µL of a 50 mM MgCl_2_ solution, 0.3 µL BioThermRed™ Taq DNA Polymerase (5 U µL^−1^) and 1.0 µL 10× buffer (including 15 mM MgCl_2_), 1.0 µL of each primer (10 mM stocks) and 1.5 µL template DNA in a total volume of 10 µL. The temperature regime consisted of initial denaturation for 3 min at 94 °C followed by 45 cycles of denaturation (94 °C for 30 sec), annealing (49 °C for 35 sec) and extension (72 °C for 1 min), with a final extension at 72 °C for 7 min.

Sanger sequencing of amplicons failed for many parasite samples because the PCR products were contaminated with amplicons derived from host DNA and from parasite pseudogenes. We therefore used Oxford Nanopore Technology (ONT) to obtain sequence reads. For this purpose, both the forward and the reverse PCR primers carried 13-bp tags (Srivathsan et al., 2019) on the 5ʹ end, resulting in sample-specific tag combinations that allowed demultiplexing of sequence reads. For sequencing library preparation, 2 µL of PCR products were pooled and the pool was purified using AMPure XP magnetic beads (Beckman Coulter, Brea, CA, USA). The concentration of the amplicon pool was measured with a Qubit 4 Fluorometer using the Qubit dsDNA HS Assay Kit (Invitrogen by Thermo Fisher Scientific, Waltham, MA, USA). Further library preparation was performed using the Ligation Sequencing Kit (SQK-LSK112 for the first batch of samples, SQK-LSK114 for the second batch of samples, both ONT), the NEBNext Ultra II End repair/dA-tailing Module (E7546, New England Biolabs) for DNA strand end repair and the NEBNext Quick Ligation Module (E6056, New England Biolabs) for adapter ligation, and followed the corresponding protocols. Sequencing of the first batch of samples (yielding 86 COI sequences) was performed on a Flongle flow cell (R9.4.1; FLO-FLG001, ONT) primed with the Flongle Flow Cell Priming Kit (EXP-FSE001, ONT); the remaining samples (yielding 50 COI sequences) were sequenced on the Flongle flow cell R10.4.1; FLO-FLG114 (ONT) primed with the Flongle Flow Cell Priming Kit (EXP-FSE002, ONT). Raw ONT data were basecalled using guppy_basecaller of Guppy version 6.4.2 with a quality threshold of five (–min_qscore 5) and the basecalling model dna_r9.4.1_450bps_sup.cfg. The ONTbarcoder software (Srivathsan et al., 2021) was used to demultiplex the sequencing reads, and AmpliconSorter (Vierstraete and Braeckman, 2022) was used to sort the demultiplexed reads by species and to obtain consensus barcode sequences for parasites and their hosts. Using the same materials as in the first batch of the present parasite sequencing (SQK-LSK112 library kit; R9.4.1, FLO-FLG001 cell; EXP-FSE001 priming kit), nanopore-based COI barcode sequences were shown to be consistent with and of the same quality as traditional Sanger sequencing (Koblmüller et al., 2024). The parasite consensus sequences were based on 9-1496 reads per sample. Only 5% of all consensuses (*n* = 136 parasite sequences) were built from less than 50 reads, and these weakly supported consensuses were identical to the most common haplotype of *P. tereticollis* in the dataset. The DNA sequence length was 655 bp. The DNA sequences were checked for compliance with the COI reading frame and blasted against GenBank for parasite species identification. The acanthocephalan COI sequences are available at GenBank (accession numbers PV792653-PV792788). Host sequences were compared to our in-lab database of local genetic lineages of *G. fossarum* (molecular operational taxonomic units, MOTUs; diBatista Borko, Sefc, *et al*., unpublished data).

In addition to the sequences generated in this study, publicly available COI sequences of *P. tereticollis* and *P. laevis* were retrieved from GenBank (accession numbers provided in Supplementary Table S3). These sequences were combined with the Nanopore-derived consensus sequences and DNA sequence alignments were performed in MEGAX vs 10.2.5 (Kumar et al., 2018). Statistical parsimony haplotype networks were plotted in PopART (Templeton et al., 1992; Leigh and Bryant, 2015) using the TCS method (Templeton et al., 1992) to visualize genetic relationships among the parasite haplotypes.

## Results

### Temporal variation in parasite prevalence

Acanthocephala prevalence was measured repeatedly, once a month from April to August and again in October, at study site #12 to assess variation over time. Prevalence estimates ranged from 7% to 9% in April (8.5%), May (7.2%) and June (9.0%), then dropped in the samples taken at the end of July (1.0%) and August (1.9%), and returned to 7% in October (6.9%). Sample sizes and number of infected amphipods are given in Table S2. At the other collection sites, prevalence was estimated only once between mid-April and early July and no decline in parasitism was observed over this period (GLM estimate = 0.01, *z* = 0.62, *P* = 0.53).

### Spatial variation in parasite prevalence

**Figure 3.**
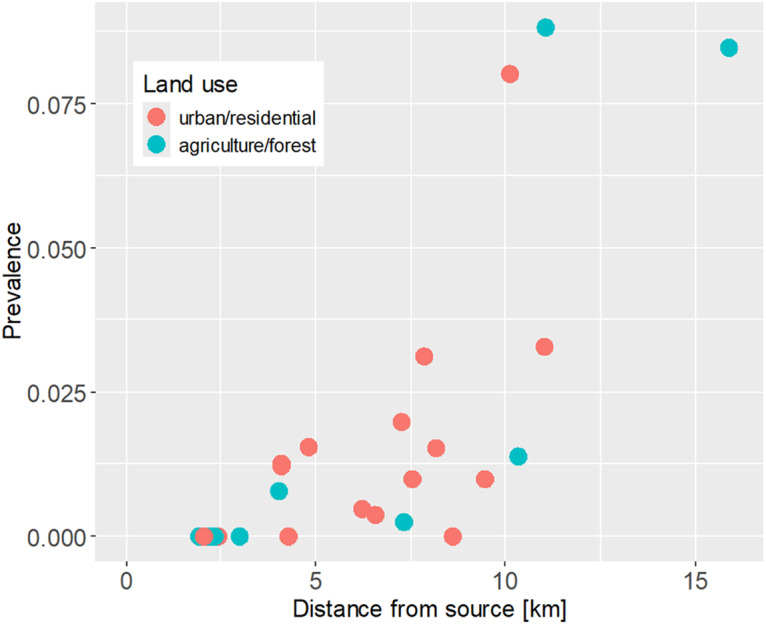
Increase of parasite prevalence with distance from stream source. Each dot represents a study site, with colours corresponding to surrounding land use.

Acanthocephala prevalence at sampling sites ranged from 0% (8 sites) to 8.8% (Table 1; Figure 2). The distribution of prevalence estimates was bimodal, with the majority of sites below 4% and three sites above 8%. The sites with the highest prevalence estimates (>8%) were located in the downstream reaches of three different streams and surrounded by both natural and artificial land surfaces. In contrast, prevalence estimates ranged from 0% to 2% in the upstream reaches, all of which were located in forested and agricultural areas. Neither surrounding land cover nor conductivity measured at the time of sampling were statistically linked to parasite prevalence, but prevalence increased significantly with distance from source (Figure 3; land cover, artificial vs natural, GLM estimate = 0.59, *z* = 1.14, *P* = 0.25; conductivity, GLM estimate = 0.0003, *z* = 0.071, *P* = 0.94; distance from source, GLM estimate = 0.36, *z* = 5.23, *P* = 1.7 × 10^−7^; VIF_(land cover)_ = 1.10, VIF_(conductivity)_ = 1.06, VIF_(distance)_ = 1.04). A model excluding the 3 sites with the highest prevalence (>8%) yielded similar results.

### Identification of parasite and host species

COI barcode sequences were successfully obtained from 136 of 154 attempted Acanthocephala samples (in the remaining cases, PCR failed or only host sequences were obtained). The majority of the sequences (*n* = 129) were identified as *P. tereticollis* by comparison to archived DNA barcodes (Figure 2). One sample was identified as *P. laevis* (Figure 2). The COI haplotype of this sample was identical to ones found previously in the River Mur in Austria (Reier et al., 2019), and clustered with the ‘eastern lineage’ of *P. laevis* (Perrot‐Minnot et al., 2018) in a network of European samples (Supplementary Fig. S1). Finally, six samples (from three sites in two streams, Figure 2) corresponded to *Polymorphus* sp. type 1 (Zittel et al., 2018), a member of the *Polymorphus minutus* species complex. With the exception of site #10, where both *Polymorphus* sp. type 1 and *P. tereticollis* were detected, only one Acanthocephala species was detected per site (Figure 2).

By sequencing the PCR products produced from the Acanthocephala DNA extracts, we also obtained the DNA barcode sequences of 129 *G. fossarum* hosts. The hosts represented five distinct mitochondrial genetic lineages (MOTUs; Table 1) across all study sites, which fell within the SEE and CEE clades defined by Wattier et al., 2020. The parasitized amphipods belonged to the three most frequent MOTUs in the study streams. No relationships between host MOTU, parasite species and parasite prevalence were apparent in the present dataset (Table 1).

### Genetic diversity of parasite COI haplotypes

**Figure 4.**
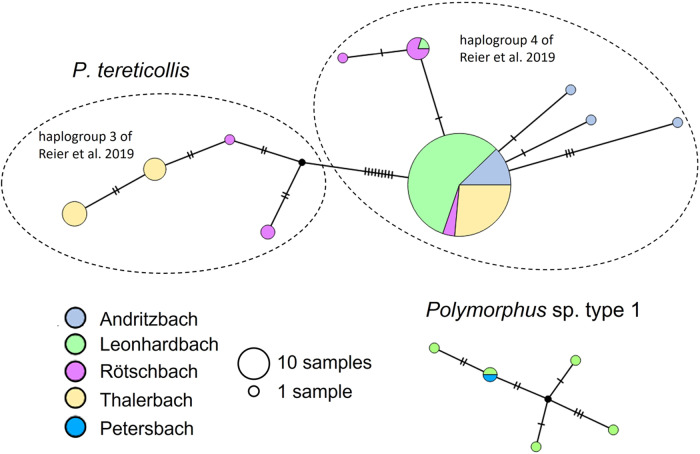
COI haplotype networks of *P. tereticollis* and *Polymorphus* sp. type 1. Colour coding and size of circles refer to stream systems and number of samples, respectively. The tick marks on the lines connecting the haplotypes indicate the number of nucleotide differences between haplotypes. In the network of *P. tereticollis*, dashed lines mark the haplogroups defined by Reier et al. (2019).

We detected 10 distinct haplotypes in *P. tereticollis* (*n* = 129 individuals; Figure 4), and the estimated haplotype diversity (H_e_) was 0.32. Two haplotype clades were separated from each other by nine mutations (1.4% divergence). One of the clades corresponds to haplogroup 3 defined by Reier et al. (2019), which comprises *P. tereticollis* collected across Europe (Supplementary Fig. S3). The other clade corresponds to haplogroup 4 (Reier et al., 2019) and has so far only been found in southeastern Austria (in the present study, and approximately 50 km further north in the River Mur, Reier et al., 2019, Supplementary Fig. S3). The most common haplotype in the present study, which was shared by the majority of individuals (67%), belonged to this clade and occurred at all sites where *P. tereticollis* were collected (Figure 4). Additional haplotypes were detected in all stream systems, with the highest diversity in the Rötschbach (*h* = 5 haplotypes; sites #1 and #2, *n* = 12 sequenced individuals), and the lowest diversity in the Leonhardbach system (*h* = 2 haplotypes; sites #9–#23, *n* = 67 sequenced individuals, Figure 4). Four of the sampling sites contained more than 1 *P. tereticollis* haplotype (#2, *h* = 4; #5, *h* = 3; #8, *h* = 4; #21, *h* = 2).

*Polymorphus* sp. type 1 (*n* = 6 individuals from three sites in two stream systems) contained five different haplotypes (H_e_ = 0.78), one of which was shared between the two stream systems in which the species was found (Figure 4).

## Discussion

### *Parasite species diversity in* G. fossarum

The prevalence of Acanthocephala at the study sites was in the single digit range. The majority of parasites extracted from the amphipod hosts were *P. tereticollis*, whose prevalence per site reached up to 8% of examined amphipods. This species has previously been shown to have a high prevalence in cyprinid and salmonid fish in the River Mur, to which the studied streams are tributaries (Reier et al., 2019). *Polymorphus* sp. type 1 (a member of the *Polymorphus minutus* species complex, Zittel et al., 2018) was found in two adjacent sites in one stream at a very low prevalence (<1%), and a single infection with *P. laevis* was detected in another stream. *P. laevis* had previously been detected in fish near the area where we conducted our study (Reier et al., 2019). The specimen collected in the course of this study shared one of the two haplotypes previously identified in the Mur (Reier et al., 2019), which fits into broader patterns of genetically structured lineages across Europe (Supplementary Fig. S1) as described by Perrot‐Minnot et al. (2018), who highlighted a strong east–west differentiation and several distinct mitochondrial clades. The lineage found in this study corresponds to the Eastern lineage, which was also detected in the Sava basin (Smrzlić et al., 2015, 2024). Genetic similarity between samples from Mur and Sava drainages (both connected to the Danube) was also observed in *P. tereticollis* (haplogroup 3; Supplementary Fig. S3).

In contrast, the present identification of *Polymorphus* sp. type 1 is the first evidence for the occurrence of this cryptic species in Austria, which was previously found in western Germany (Zittel et al., 2018). *Polymorphus* sp. type 1 has so far been shown to infect one particular genetic lineage of *G. fossarum* (type B, Zittel et al., 2018; Grabner et al., 2020). The *G. fossarum* lineage infected by *Polymorphus* sp. type 1 in the present study belongs to a different *G. fossarum* clade separated from type B by over 20 million years of divergence (Wattier et al., 2020), which suggests that *Polymorphus* sp. type 1 may be able to parasitize a broad range of *G. fossarum* types. Previous reports of *Polymorphus* cf. *minutus* in Austria refer to *Polymorphus* sp. type 3 (Jirsa et al., 2021) collected from mallards in north-eastern Austria. This cryptic species uses *G. roeselii* as intermediate host (Zittel et al., 2018) and could therefore not have been detected in the present study, in which only *G. fossarum* were collected and dissected. However, both *Gammarus* species occur in the River Mur and it remains to be tested whether *Polymorphus* sp. type 3 also occurs in south-eastern Austria.

### Parasite prevalence

The prevalence estimates in the present study are in the range of those reported from elsewhere in Europe, such as the prevalence of *P. tereticollis* and *Polymorphus minutus* in *G. fossarum* in Swiss streams (Westram et al., 2011), the prevalence of *P. tereticollis* in unidentified gammarids in French rivers (Fayard et al., 2019), and the prevalence of *Polymorphus minutus* in *G. fossarum* in two small streams in Germany (Ladewig et al., 2006). Other studies, however, have found substantially higher acanthocephalan prevalence at individual study sites (Bauer and Rigaud, 2015; Galipaud et al., 2019; Fanton et al., 2022). In Galipaud et al., 2019, *G. fossarum* were infected with *P. tereticollis, P. laevis* and *Polymorphus minutus*, and similar to our study, the highest prevalence was achieved by *P. tereticollis*.

The rarity of *P. laevis* in the studied streams is somewhat unexpected, given its presence in fish collected at Styrian sites in the Mur and its tributary, the Sulm (Reier et al., 2019). In that study, however, *P. laevis* was also detected at very low prevalence compared to *P. tereticollis*, suggesting that it plays only a minor role in the local parasite communities of the Mur and its tributaries. Similar findings have been reported elsewhere: In headwater systems of southern Germany, *P. tereticollis* was the dominant species found in salmonids, whereas the Western lineage of *P. laevis* (e.g. Perrot‐Minnot et al., 2018) occurred only in small numbers (Ros et al., 2020). The predominance of *P. tereticollis* in streams suggests that this species is more ecologically suited to colonizing rhithral environments than *P. laevis*. Support for this view also comes from intermediate host range studies: in the Weser river system (Germany), *P. laevis* was found exclusively in the native amphipod *Gammarus pulex* (which does not occur in Eastern Austria), while *P. tereticollis* showed greater flexibility in host use (Vogel and Taraschewski, 2023). Its broader host range may enable *P. tereticollis* to maintain populations and become the dominant species across a wider array of environmental and ecological conditions.

Covariation between parasite abundance and urbanization has been observed in some fish parasite systems (Hernandez et al., 2007; Taglioretti et al., 2018; Shah Esmaeili et al., 2021). In the present study, parasite prevalence did not differ significantly between sites in urban and residential areas on the one hand and sites surrounded by agricultural land and forests on the other hand. Instead, prevalence estimates increased towards downstream regions independent of surrounding land use. An exception was site #3, which was in the lower reaches of a stream close to the confluence with the river Mur, but had no infected amphipods. The stream in question is only a little more than 4 km long and likely too small to host a sufficient number of definitive hosts to establish a local parasite population. In general, the small streams in the study area are inhabited by trout, and fish also migrate from the Mur River into the lower reaches of the streams. Fish in the Mur in the study area include various cyprinids, salmonids, perch and grayling (Woschitz and Parthl, 2013). Waterfowl in the area are mainly mallards, which are common hosts of *Polymorphus minutus* (Jirsa et al., 2021) and, in smaller numbers, mergansers. Both species can occasionally be found in the small streams, including their middle and upper reaches (FG and KMS, personal observation). Therefore, possible explanations for the downstream increase in prevalence include a downstream gradient of parasite eggs due to transport in the stream current, as well as an increasing density of definitive hosts, especially fish (Blasco-Costa et al., 2013). Upstream-downstream gradients in parasite infection levels, as observed here and elsewhere (e.g. Blasco-Costa et al., 2013), are plausible but not ubiquitous. Chub (*Squalius cephalus*) sampled along a Czech river experienced lower parasite (including acanthocephalan) infections in downstream compared to upstream sites (Wenger et al., 2010), and variation in the prevalence of Acanthocephala in gammarids along a French river did not follow a longitudinal gradient but covaried with levels of environmental pollution (Fanton et al., 2022).

In addition, artificial ponds and reservoirs stocked with fish that are connected to the study streams may have contributed to elevated parasite prevalence at downstream sites in our study. For example, site #5 was located downstream of a dammed reservoir stocked with various, mostly cypriniform, fish and had the highest prevalence of *P. tereticollis* in this study. In fish ponds upstream of our sampling site #7, stocked rainbow trout, that were collected three years prior to the current study, were found to be infected with *P. tereticollis* and *P. bosniacus* (Reier et al., 2020). The two *P. tereticollis* haplotypes in the fish ponds (Reier et al., 2020) are identical to ones detected in our study, which is consistent with connectivity between pond and stream populations of *P. tereticollis*. In contrast, we did not detect *P. bosniacus* in the amphipods despite its presence in the fish pond (Reier et al., 2020). This is unlikely to be a technical artefact of DNA-based species identification, as the COI primers used in our PCR correspond to the primers used for sequencing the COI of *P. bosniacus* in Reier et al. (2020). Rather, it raises the question of whether this parasite can complete its life cycle using the local *G. fossarum* lineages as intermediate hosts. *P. bosniacus* is thought to have colonized the Danube and Rhine river systems together with its Ponto-Caspian intermediate host *Dikerogammarus villosus* (David et al., 2018; Hohenadler et al., 2018; Vogel and Taraschewski, 2023; note that initially, no distinction was made between *P. laevis* and *P. bosniacus*). This aligns with findings from the Weser river system, where *P. bosniacus* was found exclusively in *D. villosus*, despite extensive sampling of native gammarid species (Vogel and Taraschewski, 2023). Such host specificity likely prevents *P. bosniacus* from establishing in regions lacking *D. villosus*, like our study area.

Variability in parasitism rates suffered by amphipods may generally be related to species-specific parasite–host relationships. In gammarids, acanthocephalan prevalence has been shown to differ not only among recognized species but also among morphologically cryptic, yet genetically highly divergent lineages (molecular operation taxonomic units, MOTUs) within, for example, *G. fossarum* and *G. pulex* (Westram et al., 2011; Bauer and Rigaud, 2015; Zittel et al., 2018; Galipaud et al., 2019). The study sites in the present study are inhabited by five different MOTUs of *G. fossarum*, three of which were common and infected by Acanthocephala. None of the *G. fossarum* MOTUs found to be infected in our analyses were included in previous studies, and our data therefore extend the known host range of *P. tereticollis* and *Polymorphus* sp. type 1 to include these MOTUs. Unfortunately, the low parasite prevalence at sites with more than one *G. fossarum* MOTU precluded statistical tests for MOTU-specific infection patterns.

### Temporal variation in parasite prevalence

Temporal fluctuations in acanthocephalan prevalence levels have been documented in several Acanthocephala-gammarid systems, such as *P. laevis* in *G. balcanicus* (Dudiňák and Špakulová, 2003) and *G. fossarum* (van Maren, 1979) and *Polymorphus minutus* in *G. roeselii* (Médoc and Beisel, 2009) and *G. lacustris* (Spencer, 1974), but the patterns vary. Spencer (1974) described a decrease of the prevalence of *Polymorphus minutus* coinciding with the emergence of juvenile amphipods (*G. lacustris*) in the summer months, and suggested that the reduction of prevalence estimates was due to a dilution of the population with young, not visibly infected amphipods. This explanation may also apply to what we observed at site #12 in our study. The mean weight of the amphipods collected there ranged from 18 to 27 mg (max. 57 mg for the heaviest individual) in the months when the prevalence was between 7% and 9%, but was only 12–13 mg (max. 54 mg) in the two summer months when the prevalence was below 2%, indicating that the summer samples contained more small amphipods than those collected in the spring months. Long-term monitoring in several streams is required to determine whether the observed drop in visible infections during the summer months is a general, seasonal pattern in the study region.

### Intraspecific genetic diversity

We detected high genetic diversity among the small number of *Polymorphus* sp. type 1 individuals that were collected in this study. In one stream reach (sites # 10 and 11), each of five individuals had a different haplotype. This suggests that, despite low local abundances, a large effective population size might be maintained by the transport of parasites by their mobile avian hosts. In contrast, haplotype diversity in *P. tereticollis* was lower, especially in relation to sample size. Nucleotide diversity within stream systems, driven by the presence of less common haplotypes in addition to the most common one, varied independently of the level of parasite prevalence and may be related to gene flow mediated by the host fish. Most individuals of *P. tereticollis* shared a common haplotype that was present at all sampling sites. The predominance of a single haplotype at all sampling sites may indicate a recent, perhaps postglacial, population expansion, as has also been inferred on a larger geographic scale for *P. tereticollis* throughout the western Palearctic (Perrot‐Minnot et al., 2018). Although some sampling sites contained private haplotypes, no geographic structure was apparent within the study region. At a European scale, the restriction of haplogroup 4 to the Styrian part of the Mur and its tributaries indicates phylogeographic structure within *P. tereticollis*, which is, however, diluted by the widespread occurrence of haplogroup 3 across Europe (from the United Kingdom to Slovakia, and including the present study region; Supplementary Fig. S3).

## Conclusions

In conclusion, we report species-specific abundance and intraspecific genetic diversity of three species of Acanthocephala in gammarid hosts in and around an urban centre, including the first record of a recently identified cryptic species, *Polymorphus* sp. type 1 (Zittel et al., 2018), in Austria. Our data did not support a correlation between acanthocephalan infections in amphipods and urban land use. The overall low prevalence of parasites in the amphipods, along with higher values at more downstream sites, suggests that the abundance of the definitive hosts (fish and waterfowl) may be a limiting factor for Acanthocephala populations in the small streams, which may attenuate potential impacts of urbanization. While within-stream comparisons are desirable to allow some control of among-stream variation, disentangling natural and anthropogenic causes of the downstream accumulation of parasites may prove difficult in short streams, where anthropogenic disturbances are often concentrated in the downstream reaches.

Our study demonstrates the usefulness of collecting infected amphipods to obtain Acanthocephala samples suitable for, e.g. genetic analysis. Importantly, given the limited mobility of amphipods (Alther et al., 2021; Švara et al., 2022; Weiss et al., 2022), the Acanthocephala collected in this way represent the natal population, i.e., individuals that developed from eggs that were released in the same stream. This allows for a more spatially explicit analysis of parasite population structure than sampling from the definitive hosts, which may include individuals migrating through the study area.

## Supporting information

Gallhammer et al. supplementary material Gallhammer et al. supplementary material

## Data Availability

DNA sequences generated in this study are available at GenBank (accession numbers PV792653-PV792788). All other data used in the study are included in the submission.

## Supplementary material

The supplementary material for this article can be found at https://doi.org/10.1017/S0031182025100449.
