# Supplementary material for: Prevalence and diversity of Acanthocephala in stream-dwelling amphipods (Gammarus fossarum) around an urban area in the eastern Alpine foothills
Source: Parasitology. 2025 Jul 3;152(7):657–67. doi: 10.1017/S0031182025100449 (PMC12418285; doi:10.1017/S0031182025100449)
Supplement: Gallhammer et al. supplementary material [file S0031182025100449sup001.docx]

Supplementary material

Prevalence and diversity of Acanthocephala in stream-dwelling amphipods (*Gammarus fossarum*) around an urban area in the eastern Alpine foothills

Fabian Gallhammer^1^, Jacqueline Grimm^1^, Susanne Reier^2^, Kristina M. Sefc^1^

^1^ Institute of Biology, University of Graz, Graz, Austria

^2^ First Zoological Department, Natural History Museum Vienna, Vienna, Austria

Supplementary Fig. S1. Median-joining haplotype network of *Pomphorhynchus laevis* based on partial COI sequences. The network distinguishes between the Eastern and Western mitochondrial lineages of *P. laevis*, as described by Perrot-Minnot et al. (2018) and Reier et al. (2019). Haplotype relationships are shown across multiple European river systems. The single *P. laevis* sample from this study (Austria, Mur River) clusters within the Eastern lineage, along with haplotypes previously found in the Sava River drainage.

GenBank accession numbers and country of origin of COI sequences in the network are given in table S3.


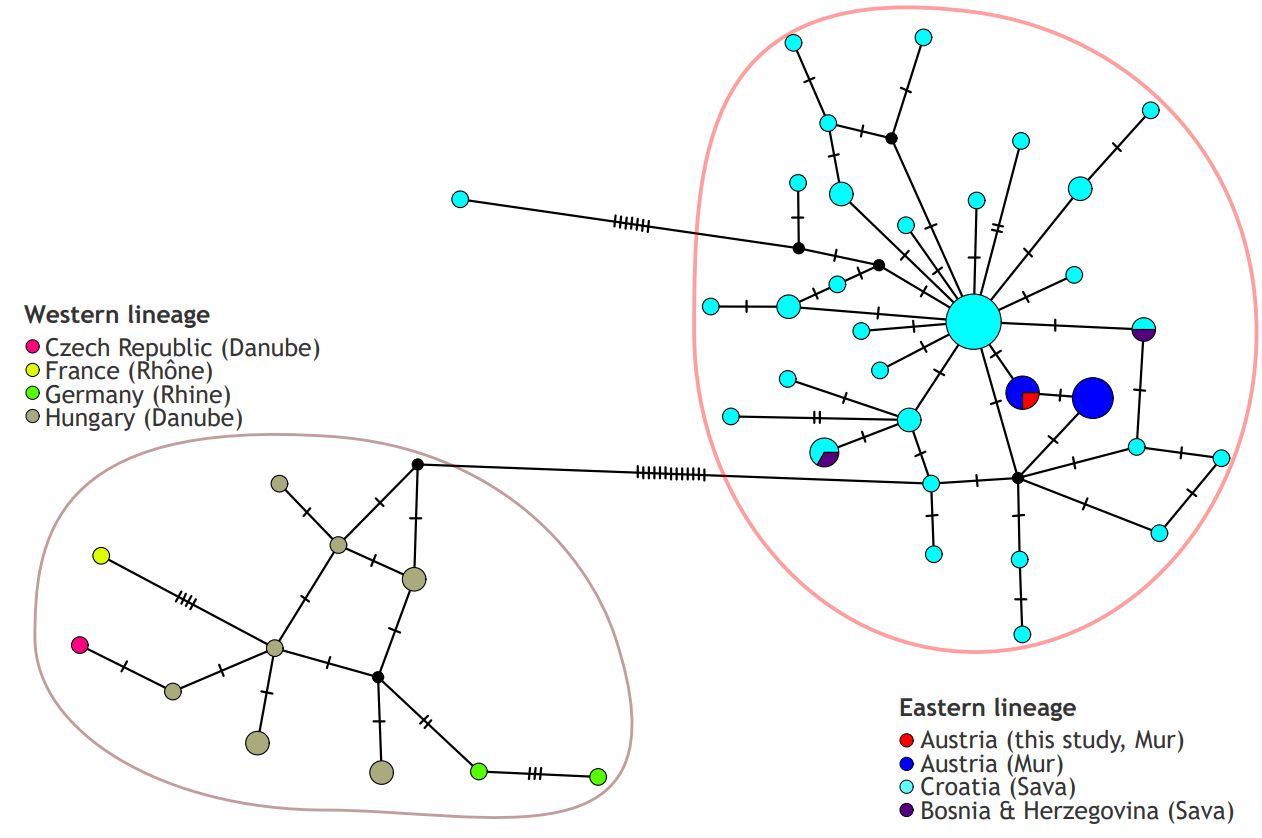


Supplementary Fig. S2. Median-joining haplotype network of Polymorphus spp. based on mitochondrial COI sequences. Three distinct genetic clusters are evident and correspond to Polymorphus sp. type 1, type 2, and type 3 (Zittel et al., 2018), associated with different amphipod and bird hosts as indicated. Each circle represents a unique haplotype, with the size of the circle proportional to the number of individuals sharing that haplotype. The small black lines between haplotypes indicate mutational steps; grey circles indicate haplotypes missing in the study sampling. Colors correspond to sampling locations.

The sequences were obtained from the BOLD project ‘PACDE’ (Zittel et al., 2018) and GenBank (accession numbers MT184812- MT184814; Jirsa et al., 2021).


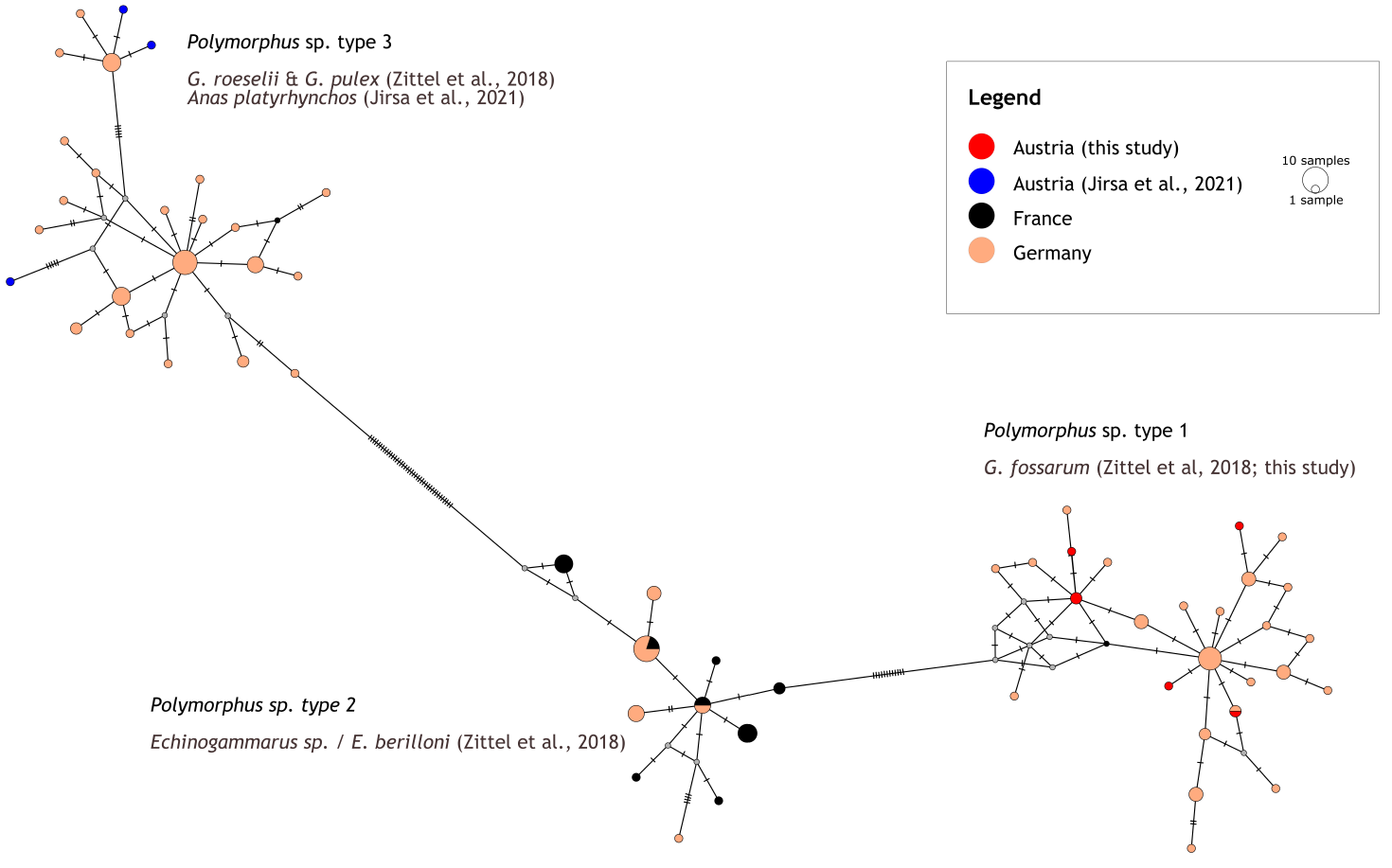


Supplementary Fig. S3. Median-joining haplotype network of *Pomphorhynchus tereticollis* based on partial COI sequences. The network reflects the four major haplogroups (Groups 1–4) proposed by Reier et al. (2019). Samples from this study are found in Group 3 and Group 4. Group 3 includes haplotypes from a wide geographic range, while group 4 appears to be geographically restricted and has thus far been detected exclusively in the Mur River system in Austria.

GenBank accession numbers and country of origin of COI sequences in the network are given in table S3.


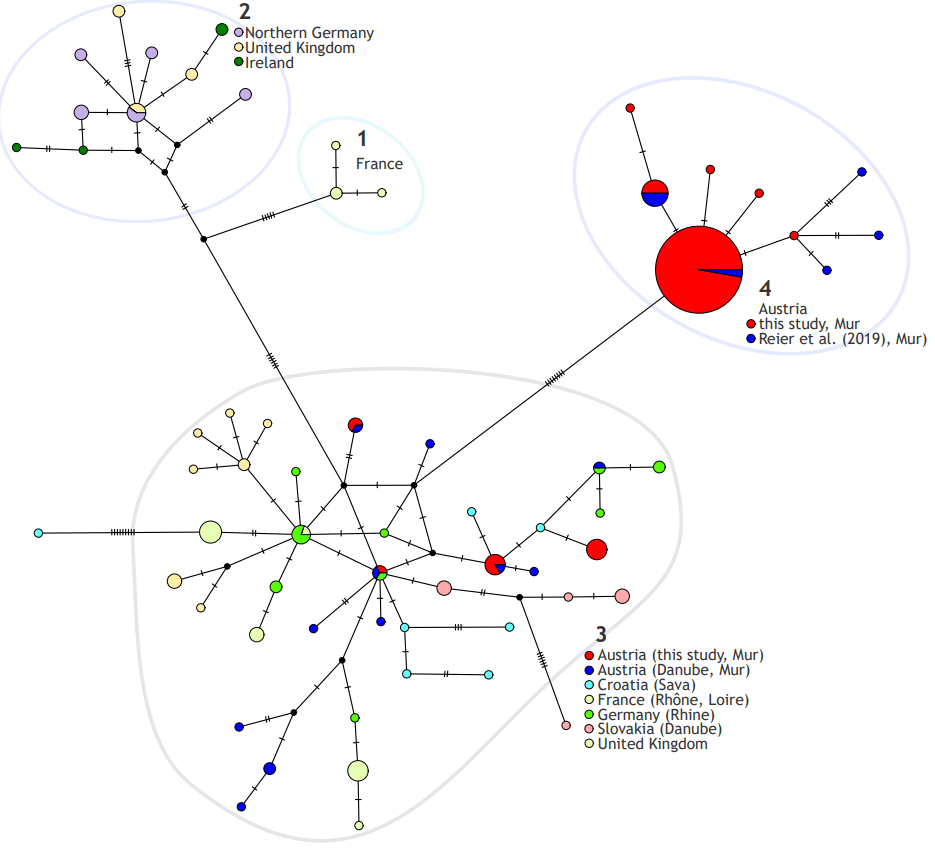


Supplementary Table S1. Characteristics of the sampling sites. This table complements the information given Table 1.

| **site** | **date** | **longitude** | **latitude** | **distance from source (km)** | **T (°C)** | **oxygen (%)** | **mean weight, parasitized amphipods (mg)** | **mean weight, non-parasitized amphipods (mg)** |
| --- | --- | --- | --- | --- | --- | --- | --- | --- |
| **1** | 05.07.2023 | 15.375716 | 47.180402 | 10.32 | 12.8 | 103 | 20.33 | 12.40 |
| **2** | 21.06.2023 | 15.32285 | 47.156208 | 15.88 | 19.6 | 90 | 33.13 | 42.34 |
| **3** | 21.06.2023 | 15.349572 | 47.125026 | 4.26 | 16.8 | 84 | NA | 15.36 |
| **4** | 30.05.2023 | 15.364876 | 47.061282 | 7.33 | 14.6 | 78 | 28.00 | 26.14 |
| **5** | 30.05.2023 | 15.374423 | 47.088965 | 11.04 | 15.6 | 85 | 22.48 | 24.31 |
| **6** | 01.06.2023 | 15.418931 | 47.144456 | 2.43 | 12.2 | 83 | NA | 23.97 |
| **7** | 25.05.2023 | 15.42692 | 47.127741 | 4.80 | 10.9 | 85 | 14.25 | 17.28 |
| **8** | 23.05.2023 | 15.414559 | 47.104228 | 8.18 | 12.4 | 84 | 11.63 | 18.70 |
| **9** | 04.05.2023 | 15.510161 | 47.120152 | 2.19 | 11.8 | 78 | NA | 17.95 |
| **10** | 03.05.2023 | 15.475272 | 47.098944 | 7.53 | 11.9 | 97 | 22.00 | 28.39 |
| **11** | 27.04.2023 | 15.460769 | 47.090963 | 9.46 | 11.3 | 91 | 14.75 | 31.87 |
| **12** | 21.04.2023, 11.05.2023, 14.06.2023 | 15.46415 | 47.075769 | 10.11 | 12.5 | 84 | 16.73 | 24.20 |
| **13** | 19.06.2023 | 15.456825 | 47.071486 | 11.01 | 19.5 | 82 | 19.92 | 13.51 |
| **14** | 04.05.2023 | 15.521654 | 47.111737 | 2.33 | 11.9 | 76 | NA | 27.79 |
| **15** | 04.05.2023 | 15.510857 | 47.101474 | 4.03 | 10.6 | 79 | 34.20 | 42.01 |
| **16** | 26.04.2023 | 15.492236 | 47.09126 | 6.56 | 10 | 84 | 26.00 | 37.23 |
| **17** | 26.04.2023 | 15.472621 | 47.084636 | 8.60 | 9.2 | 90 | NA | 24.52 |
| **18** | 20.04.2023 | 15.546876 | 47.081004 | 1.93 | 7.6 | 76 | NA | 31.53 |
| **19** | 20.04.2023 | 15.533976 | 47.075769 | 2.99 | 10.7 | 85 | NA | 27.48 |
| **20** | 20.04.2023 | 15.52186 | 47.075149 | 4.11 | 11 | 87 | 20.74 | 23.43 |
| **21** | 21.04.2023 | 15.497839 | 47.076665 | 6.23 | 10.6 | 94 | 26.35 | 22.57 |
| **22** | 24.04.2023 | 15.485316 | 47.076854 | 7.27 | 10.1 | 87 | 16.80 | 20.50 |
| **23** | 24.04.2023 | 15.478572 | 47.07613 | 7.86 | 10.4 | 93 | 16.92 | 20.65 |
| **24** | 03.07.2023 | 15.471665 | 47.049402 | 4.08 | 20.3 | 127 | 12.50 | 8.77 |
| **25** | 15.06.2023 | 15.484376 | 47.059163 | 2.06 | 14.2 | 76 | NA | 14.95 |

Supplementary Table S2. Parasite prevalence and conditions during the sampling at site #12 from April to October 2023. Sample size refers to the number of amphipods examined, and ‘No. inf.’ is the number of infected amphipods. Further information on site #12 is given in Table 1 and Table S1.

| **site** | **date** | **sample size** | **No. inf.** | **prevalence (%)** | **T (°C)** | **cond. (μS/cm)** | **oxygen (%)** | **mean weight, parasitized amphipods (mg)** | **mean weight, non-parasitized amphipods (mg)** |
| --- | --- | --- | --- | --- | --- | --- | --- | --- | --- |
| 12 | 21.04.2023 | 59 | 5 | 0.085 | 11.3 | NA | 87 | 13.08 | 17.92 |
| 12 | 11.05.2023 | 320 | 23 | 0.072 | 11.5 | 540 | 82 | 16.70 | 27.97 |
| 12 | 14.06.2023 | 233 | 21 | 0.090 | 14.7 | 354 | 82 | 20.43 | 26.72 |
| 12 | 26.07.2023 | 385 | 4 | 0.010 | 17.7 | 406 | 98 | 1.75 | 12.95 |
| 12 | 22.08.2023 | 375 | 7 | 0.019 | 22.1 | 526 | 101 | 9.86 | 11.77 |
| 12 | 17.10.2023 | 639 | 44 | 0.069 | 9.5 | 562 | 111 | 6.64 | 10.24 |

Supplementary Table S3. GenBank accession numbers and country of origin of COI sequences of *P. tereticollis* and *P. laevis*, which were used to supplement the data generated in the current study to construct the haplotype networks in Fig. S1 and Fig. S2.

| **GenBank Accession #** | **country** |
| --- | --- |
| *Pomphorhynchus laevis* |  |
| MK612529 | Austria |
| MK612526 | Austria |
| MK612530 | Austria |
| MK612528 | Austria |
| MK612531 | Austria |
| MK133343 | Austria |
| MK612525 | Austria |
| MK612527 | Austria |
| MK612532 | Austria |
| MK612524 | Austria |
| MK133344 | Bosnia & Herzegovina |
| KF559286 | Croatia |
| KF559296 | Croatia |
| KF559284 | Croatia |
| KF559290 | Croatia |
| KF559300 | Croatia |
| KF559291 | Croatia |
| KF559292 | Croatia |
| KF559298 | Croatia |
| KF559294 | Croatia |
| KF559299 | Croatia |
| KF559289 | Croatia |
| KF559295 | Croatia |
| KF559285 | Croatia |
| KF559288 | Croatia |
| OQ569505 | Croatia |
| OQ569515 | Croatia |
| OQ569507 | Croatia |
| OQ569517 | Croatia |
| OQ569512 | Croatia |
| OQ569499 | Croatia |
| OQ569496 | Croatia |
| OQ569518 | Croatia |
| OQ569494 | Croatia |
| OQ569495 | Croatia |
| OQ569510 | Croatia |
| OQ569497 | Croatia |
| OQ569502 | Croatia |
| OQ569498 | Croatia |
| OQ569490 | Croatia |
| OQ569506 | Croatia |
| OQ569491 | Croatia |
| OQ569509 | Croatia |
| OQ569516 | Croatia |
| OQ569514 | Croatia |
| OQ569501 | Croatia |
| OQ569508 | Croatia |
| OQ569519 | Croatia |
| OQ569500 | Croatia |
| OQ569511 | Croatia |
| OQ569492 | Croatia |
| OQ569520 | Croatia |
| OQ569503 | Croatia |
| OQ569513 | Croatia |
| OQ569504 | Croatia |
| OQ569493 | Croatia |
| EF051071 | Czech Republic |
| EF051064 | France |
| MT216153 | Germany |
| MT216152 | Germany |
| AY423349 | Hungary |
| AY423350 | Hungary |
| AY423348 | Hungary |
| EF051069 | Hungary |
| EF051066 | Hungary |
| EF051065 | Hungary |
| EF051068 | Hungary |
| EF051062 | Hungary |
| EF051067 | Hungary |
| EF051070 | Hungary |
| *Pomphorhynchus tereticollis* |  |
| MK612534 | Austria |
| MK612545 | Austria |
| MK612535 | Austria |
| MK612536 | Austria |
| MK612537 | Austria |
| MK612538 | Austria |
| MK612539 | Austria |
| MK612540 | Austria |
| MK612541 | Austria |
| MK612542 | Austria |
| MK612543 | Austria |
| MK612533 | Austria |
| MK612544 | Austria |
| MN780983 | Austria |
| MN780978 | Austria |
| MN780980 | Austria |
| MN780982 | Austria |
| MN780986 | Austria |
| MN780984 | Austria |
| MN780981 | Austria |
| MN780985 | Austria |
| OQ569526 | Croatia |
| OQ569524 | Croatia |
| OQ569525 | Croatia |
| OQ569522 | Croatia |
| OQ569523 | Croatia |
| OQ569528 | Croatia |
| OQ569529 | Croatia |
| OQ569527 | Croatia |
| LN994969 | France |
| LN994968 | France |
| LN994967 | France |
| LN994966 | France |
| LN994965 | France |
| LN994964 | France |
| LN994963 | France |
| LN994962 | France |
| LN994961 | France |
| LN994960 | France |
| LN994959 | France |
| LN994958 | France |
| LN994957 | France |
| LN994956 | France |
| LN994955 | France |
| LN994954 | France |
| LN994953 | France |
| LN994952 | France |
| LN994951 | France |
| AY423351 | France |
| AY423352 | France |
| AY423353 | France |
| LN994978 | Germany (Northern) |
| LN994977 | Germany (Northern) |
| LN994976 | Germany (Northern) |
| LN994975 | Germany (Northern) |
| LN994974 | Germany (Northern) |
| LN994973 | Germany (Northern) |
| LN994972 | Germany (Northern) |
| JN695507 | Germany (Northern) |
| JN695506 | Germany (Northern) |
| JF706706 | Germany (Northern) |
| JN695508 | Germany (Northern) |
| JN695505 | Germany (Northern) |
| LN994971 | Germany (Rhine) |
| LN994970 | Germany (Rhine) |
| MT216163 | Germany (Rhine) |
| MT216167 | Germany (Rhine) |
| MT216160 | Germany (Rhine) |
| MT216170 | Germany (Rhine) |
| MT216171 | Germany (Rhine) |
| MT216159 | Germany (Rhine) |
| MT216165 | Germany (Rhine) |
| MT216172 | Germany (Rhine) |
| MT216162 | Germany (Rhine) |
| MT216164 | Germany (Rhine) |
| MT216161 | Germany (Rhine) |
| MT216166 | Germany (Rhine) |
| MT216169 | Germany (Rhine) |
| MT216168 | Germany (Rhine) |
| LN994998 | Ireland |
| LN994997 | Ireland |
| LN994996 | Ireland |
| LN994995 | Ireland |
| MT682955 | Ireland |
| MT682954 | Ireland |
| MT682956 | Ireland |
| LN994986 | Slovakia |
| LN994985 | Slovakia |
| LN994984 | Slovakia |
| LN994983 | Slovakia |
| LN994982 | Slovakia |
| LN994981 | Slovakia |
| LN994980 | Slovakia |
| LN994979 | Slovakia |
| LN995000 | United Kingdom |
| LN994999 | United Kingdom |
| LN994994 | United Kingdom |
| LN994993 | United Kingdom |
| LN994992 | United Kingdom |
| LN994991 | United Kingdom |
| LN994990 | United Kingdom |
| LN994989 | United Kingdom |
| LN994988 | United Kingdom |
| KY075795 | United Kingdom |
| KY075799 | United Kingdom |
| KY075800 | United Kingdom |
| KY075798 | United Kingdom |
| KY075796 | United Kingdom |
| KY075794 | United Kingdom |
| KY075797 | United Kingdom |

**References:**

Jirsa, F, Reier, S and Smales, L (2021) Helminths of the mallard Anas platyrhynchos Linnaeus, 1758 from Austria, with emphasis on the morphological variability of Polymorphus minutus Goeze, 1782. *Journal of Helminthology* 95, e16.

Perrot‐Minnot, M-J, Špakulová, M, Wattier, R, Kotlik, P, Düşen, S, Aydoğdu, A and Tougard, C (2018) Contrasting phylogeography of two Western Palaearctic fish parasites despite similar life cycles. *Journal of Biogeography* 45, 101–115.

Reier, S, Sattmann, H, Schwaha, T, Harl, J, Konecny, R and Haring, E (2019) An integrative taxonomic approach to reveal the status of the genus Pomphorhynchus Monticelli, 1905 (Acanthocephala: Pomphorhynchidae) in Austria. *International Journal for Parasitology: Parasites and Wildlife* 8, 145–155.

Zittel, M, Grabner, D, Wlecklik, A, Sures, B, Leese, F, Taraschewski, H and Weigand, AM (2018) Cryptic species and their utilization of indigenous and non-indigenous intermediate hosts in the acanthocephalan Polymorphus minutus sensu lato (Polymorphidae). *Parasitology* 145, 1421–1429.
